# Copper(II)-binding equilibria in human blood

**DOI:** 10.1038/s41598-020-62560-4

**Published:** 2020-03-30

**Authors:** Tiina Kirsipuu, Anna Zadorožnaja, Julia Smirnova, Merlin Friedemann, Thomas Plitz, Vello Tõugu, Peep Palumaa

**Affiliations:** 1 Department of Chemistry and Biotechnology, Tallinn University of Technology Akadeemia tee 15, 12618 Tallinn, Estonia; 2 Wilson Therapeutics AB, Kungsgatan 3, S-111 43, Stockholm, Sweden

**Keywords:** Bioinorganic chemistry, Metals, Proteins

## Abstract

It has been reported that Cu(II) ions in human blood are bound mainly to serum albumin (HSA), ceruloplasmin (CP), alpha-2-macroglobulin (α2M) and His, however, data for α2M are very limited and the thermodynamics and kinetics of the copper distribution are not known. We have applied a new LC-ICP MS-based approach for direct determination of Cu(II)-binding affinities of HSA, CP and α2M in the presence of competing Cu(II)-binding reference ligands including His. The ligands affected both the rate of metal release from Cu•HSA complex and the value of *K*_*D*_. Slow release and *K*_*D*_ = 0.90 pM was observed with nitrilotriacetic acid (NTA), whereas His showed fast release and substantially lower *K*_*D*_ = 34.7 fM (50 mM HEPES, 50 mM NaCl, pH 7.4), which was explained with formation of ternary His•Cu•HSA complex. High mM concentrations of EDTA were not able to elicit metal release from metallated CP at pH 7.4 and therefore it was impossible to determine the *K*_*D*_ value for CP. In contrast to earlier inconclusive evidence, we show that α2M does not bind Cu(II) ions. In the human blood serum ~75% of Cu(II) ions are in a nonexchangeable manner bound to CP and the rest exchangeable copper is in an equilibrium between HSA (~25%) and Cu(II)-His-Xaa ternary complexes (~0.2%).

## Introduction

Copper is an essential cofactor for more than twenty proteins that play important roles in cellular energy production, antioxidative defense and oxidative metabolism. Organismal copper metabolism is strictly regulated and its deflections are characteristic for many diseases. Disturbance of copper metabolism is the primary cause of monogenic Wilson’s and Menkes diseases^1^ and it is also involved in progression of inflammation, cancer, atherosclerosis^2^ and many neurodegenerative diseases including the Alzheimer’s disease^3^.

The extracellular copper pool in the body is stored, transported and distributed in the body mainly by blood. According to the current opinion, the copper ions in the human blood serum are distributed between three proteins: ceruloplasmin (CP), albumin (HSA) and alpha-2-macroglobulin (α2M) accounting for approx. 70, 15 and 10% of copper, respectively^4,5^. The remaining 5% of copper is functioning as a cofactor in Cu,ZnSOD-3, clotting factors V and VIII, amine and diamine oxidases, ferroxidase II and some other enzymes^5^. Existence of a low-molecular-weight (LMW) copper pool bound with His in blood was proposed for a half of century^6,7^. However, equilibrium dialysis data^8^ and direct EPR measurements^9^ showed that HSA competes effectively with His for binding of Cu(II) at equimolar concentrations suggesting that excessive concentration of HSA in the blood serum should avoid the binding of Cu(II) to His and other free amino acids in the blood. At the same time the existence of LMW copper pool in blood serum is recently confirmed^10^ and the “saga of Cu(II)-His” in blood^11^ still continues.

Structure and functioning of major blood copper proteins have been intensively studied for many decades, however, information about their metal-binding properties is still limited and often inconsistent.

CP is a monomeric glycoprotein with MW of 132 kD^12^ and its average concentration in blood serum is 1.9 μM^13^. CP is metallated in the secretory pathway with six Cu ions bound in a highly cooperative manner^14^. It is known that the cupric ions bound to CP are “not exchangeable”^2^, which seriously complicates determination of the Cu(II)-binding affinity of CP. CP is an enzyme (EC 1.16.3.1) exhibiting oxidase activity towards Fe(II) ions and numerous aromatic compounds^5^, however, it is involved also in the transport of copper into mammalian cells^15^.

α2M is a homotetrameric glycoprotein with MW of 720 kD^16^ responsible for binding and inactivation of proteases^17^. The average concentration of α2M in human plasma is 1.3 μM and there is evidence that α2M can bind copper ions and participate in copper delivery into mammalian cells^18^. Limited information about Cu(II)-binding properties of α2M indicates that only two Cu(II) ions could be bound to tetramer with higher affinity than that of HSA^18^.

HSA is the most abundant and probably the most studied protein in the blood. The average concentration of HSA in serum is 650 μM and it constitutes more than 50% of serum proteins^19,20^. HSA (MW 66.5 kD) is a monomeric multicargo transport and storage protein containing seven hydrophobic binding pockets for organic molecules such as fatty acids, metabolites, hormones and drugs^21,22^. *In vitro* studies have identified also four distinct metal binding sites in HSA, differing in structure and metal-binding specificity^23^. The most studied metal-binding site of HSA is the N-terminal site (NTS)^24^ also known as ATCUN^25^, which binds Cu(II) ions in physiological conditions. The second site is called Multi-Metal Binding Site (MBS), responsible mainly for binding of Zn(II) ions^23^. The third site is composed from a free Cys34 residue and its surrounding and the fourth site with unknown location is called Site B. Physiological relevance of last two sites is unknown, however, they might interact with toxic metal ions like Cd(II) or Ni(II) or with metallodrugs^23^.

The NTS, includes three N-terminal amino acid residues and is characterized by the presence of His in the third position^24^. Only a small fraction (~2%) of these sites are occupied by Cu(II) ions in physiological conditions^23^, however, binding and concomitant redox silencing of pro-oxidant Cu(II) ions by HSA gives an important contribution to the antioxidative capacity of the blood^26^.

Cu(II)-binding properties of NTS have been in the focus of intensive studies. The dissociation constant (*K*_*D*_) values for Cu(II) in NTS of HSA (Asp-Ala-His) and of bovine serum albumin (Asp-Thr-His), have been determined by a variety of methods such as potentiometric titration, equilibrium dialysis, ultrafiltration, isothermal titration calorimetry, Cu(II) electrode and several spectroscopic methods, including EPR^27^. However, the estimated values for the conditional dissociation constant vary from picomolar (log *K*_*D*_ = −11.18) to subfemtomolar (log *K*_*D*_ = −16.18) range^28,23^. The methods used for the study of the binding of Cu(II) to HSA and other Cu(II) proteins so far rely on indirect estimation of the equilibrium concentration of Cu•Protein complex, which can lead to misinterpretation of experimental results^29^. Such problems can be avoided by using methods, which can directly determine the equilibrium concentrations of the metallated complex.

The aim of the current study was to estimate the Cu(II)-binding affinities of major serum copper proteins (HSA, CP and α2M) in comparison with LMW Cu(II)-binding reference ligands including His by using an unified and direct approach, which gives thermodynamic background for understanding the distribution copper in the blood. For this purpose we have elaborated a new LC-ICP MS based approach for direct detection of Cu•Protein complex in the presence of metal-competing LMW Cu(II)-binding ligands. To cover the wide range of metal-binding affinities of different proteins high-affinity DTPA and EDTA, intermediate affinity NTA and low-affinity His were used in titrations. The kinetics of the metal release from Cu•Protein complex was also studied to ensure that the equilibrium was reached. In result comparable Cu(II)-binding properties of HSA, CP and α2M have been determined and an accelerating effect of His on metal release from HSA has been described. Results enable systematical and quantitative overview from the copper-binding equilibria in human blood.

## Results

### Elaboration of LC-ICP MS for determination of HSA interaction with Cu(II) ions

**Figure 1:**
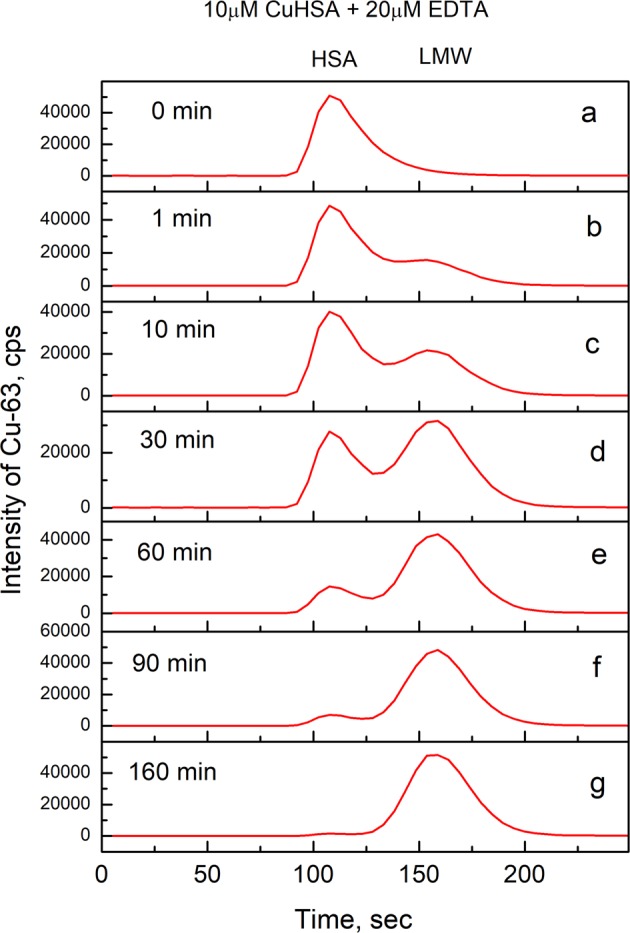
Demetallation of Cu•HSA by EDTA monitored by LC-ICP MS. Conditions: 10 μM Cu•HSA, 20 μM EDTA; incubation buffer 50 mM HEPES, 50 mM NaCl, pH 7.4; incubation times: (**a**) - 0 min, (**b**) - 1 min, (**c**) −10 min, (**d**) - 30 min, (**e**) - 60 min, (**f**) - 90 min, (**g**) - 160 min; column - 1 ml Sephadex G25 Superfine; elution buffer 200 mM NH_4_NO_3_, pH 7.4; flow rate 0.4 ml/min; injection volume 10 μl. Cu-63 was monitored by ICP MS. Figure was created by program Origin 9 Pro (https://www.originlab.com/).

The high and low-molecular weight (HMW and LMW) copper pools were separated by size-exclusion chromatography (SEC) on a 1 ml Sephadex G25 Superfine column. The separation of HMW and LMW compounds was completed in 4 minutes (flow rate 0.4 ml/min). When an equimolar amount of Cu(II) was added to 10 μM HSA all copper was found in the HMW peak (Fig. 1a). Thus, the kinetic stability of Cu•HSA complex is sufficient for SEC separation.

Adding of Cu(II) to HSA in a twofold excess did not increase the amount of copper in the HMW peak, which confirms that only the binding to the single high-affinity site is seen in the experiment. At the same time, the peak of LMW copper was not observed in the chromatogram, demonstrating that the column material has some capacity to bind the “free” Cu(II) ions. We confirmed that the column-bound copper could be quantitatively removed from the column by a subsequent elution with 10 μl of 1 mM EDTA. In the Cu(II)-competition experiments with LMW ligands like EDTA, NTA, DTPA, the copper was quantitatively eluted from the column, suggesting that the binding affinity of column matrix is sufficiently lower as that for listed ligands. In case of HSA - His competition experiments LMW copper peak was smaller than expected at lower His concentrations. To quarantee similar LC conditions for all experiments, column decoppering with10 μl of 1 mM EDTA was performed before each LC ICP MS experiment.

### Demetallation of Cu(II)•HSA with copper-binding ligands

**Figure 2:**
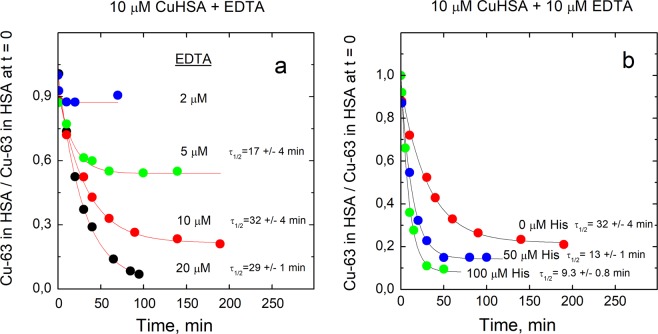
Demetallation kinetics of Cu•HSA by EDTA in the absence and presence of His. Decrease of relative content of Cu-63 in HSA in time (**a**); effect of His on demetallatiuon of Cu•HSA by EDTA (**b**). Conditions: 10 μM Cu•HSA, 2–20 μM EDTA (**a**); 10 μM Cu•HSA, 10 μM EDTA, 0–100 μM His (**b**); incubation buffer 50 mM HEPES, 50 mM NaCl, pH 7.4; LC-ICP MS: column - 1 ml Sephadex G25 Superfine; elution buffer 200 mM NH_4_NO_3_, pH 7.4; flow rate 0.4 ml/min; injection volume 10 μl, Cu-63 was monitored by ICP MS. Figure was created and fittings were performed by program Origin 9 Pro (https://www.originlab.com/).

Incubation of Cu•HSA complex with EDTA decreased the HMW copper peak and increased the LMW copper peak (Fig. 1b–g). The half-life of Cu•HSA demetallation in the presence of EDTA was 20–30 min (Fig. 2) and the protein was almost fully demetallated already by an equimolar amount of EDTA. Thus, the dissociation of Cu(II) ions from HSA in the presence of EDTA is relatively slow and the affinity of Cu(II) for HSA is lower than that for EDTA. EDTA binds one Cu(II) ion with *K*_*D*_ = 1.26 × 10^−16^ M at pH 7.4^30^.

**Figure 3:**
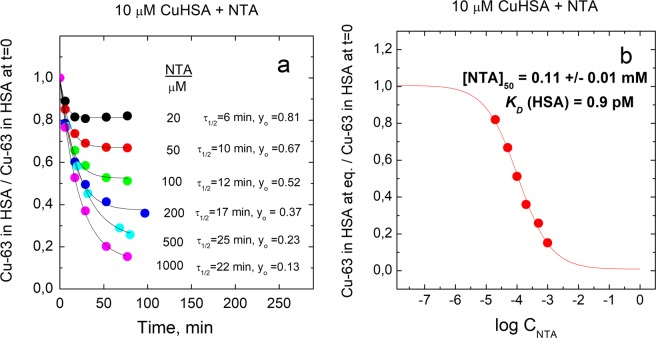
Demetallation kinetics of Cu•HSA by NTA and determination of dissociation constant for Cu•HSA. Decrease of relative content of Cu-63 in HSA in time (**a**); relative content of Cu-63 in HSA at equilibrium at different concentrations of NTA (**b**). Conditions: 10 μM Cu•HSA, 10–1000 μM NTA; incubation buffer 50 mM HEPES, 50 mM NaCl, pH 7.4; LC-ICP MS: column - 1 ml Sephadex G25 Superfine; elution buffer 200 mM NH_4_NO_3_, pH 7.4; flow rate 0.4 ml/min; injection volume 10 μl, Cu-63 was monitored by ICP MS. Figure was created and fittings were performed by program Origin 9 Pro (https://www.originlab.com/).

**Figure 4:**
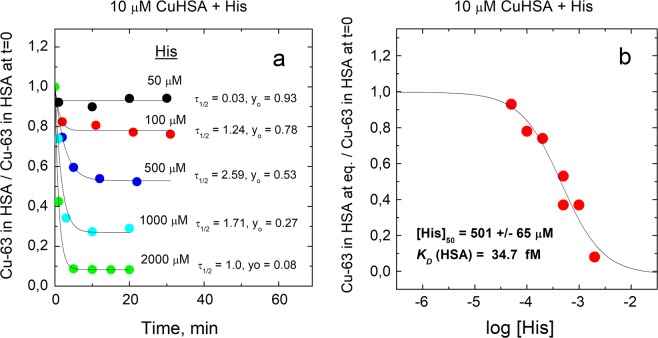
Demetallation kinetics of Cu•HSA by His and determination of dissociation constant for Cu•HSA. Decrease of relative content of Cu-63 in HSA in time (**a**); relative content of Cu-63 in HSA at equilibrium at different concentrations of His (**b**). Conditions: 10 μM Cu•HSA, 10–2000 μM His; incubation buffer 50 mM HEPES, 50 mM NaCl, pH 7.4; LC-ICP MS: column - 1 ml Sephadex G25 Superfine; elution buffer 200 mM NH_4_NO_3_, pH 7.4; flow rate 0.4 ml/min; injection volume 10 μl, Cu-63 was monitored by ICP MS. Figure was created and fittings were performed by program Origin 9 Pro (https://www.originlab.com/).

A substantially weaker Cu(II)-binding ligand NTA (*K*_*D*_ = 1.99 × 10^−11^ M at pH = 7.4^31^) also demetallated Cu•HSA with the half-life of 20–25 min. As expected, a substantially higher than equimolar concentration of NTA was required for complete demetallation of HSA (Fig. 3a), which allows to determine the *K*_*D*_ value for Cu•HSA complex. From the fractional content of Cu•HSA at equilibrium in the presence of different concentrations of NTA (Fig. 3b) the *K*_*D*_ value equal to 0.900 ± 0.091 pM was determined by using Eqn. 4 as described in Experimental section. Demetallation of Cu•HSA complex in the presence of His was also studied. His forms 2:1 complex with Cu(II) at pH = 7.4 where *K*_*D*1_ = 3.7 × 10^−9^ M and *K*_*D*2_ = 4.7 × 10^−7^ M^31^. In contrast to EDTA and NTA, His demetallated Cu•HSA with fast kinetics (half-life of appr. 1-2 min) and at relatively high concentrations (Fig. 4a), which also enables determination of *K*_*D*_ for Cu•HSA complex. By using of fractional content of Cu•HSA at equilibrium conditions at different concentrations of His (Fig. 4b) the *K*_*D*_ = 34.7 ± 4.5 fM was determined according to the Eqn. 4 as described in Experimental section.

A separate experiment showed that low concentrations of His that alone did not demetallate Cu•HSA, substantially increased the rate of the HSA demetallation by EDTA: three and sixfold increases were observed in the presence of 50 μM and 100 μM of His, respectively (Fig. 2b). 0.5 mM Glu and Gly were not able to demetallate Cu•HSA complex and did not accelerate its demetallation in the presence of EDTA (Data not shown).

### Attempts to demetallate Cu•CP with copper-binding ligands

In Cu•CP samples the copper eluted from the Sephadex G25 column in HMW region confirming that metal is strongly bound to the protein. Adding up to 200 mM of strong Cu(II) chelators (DTPA, EDTA) at pH 7.4 did not decrease the metal content in Cu•CP peak even after 100 min incubation with 200 mM EDTA or 21 h incubation with 100 mM DTPA (Supplementary Information, Figure S1a). At high, non-physiological pH values (pH 11) Cu•CP could be demetallated by EDTA in a dose-dependent manner (Supplementary Information, Figure S1b).

### Metallation of α2M with Cu(II) ions

**Figure 5:**
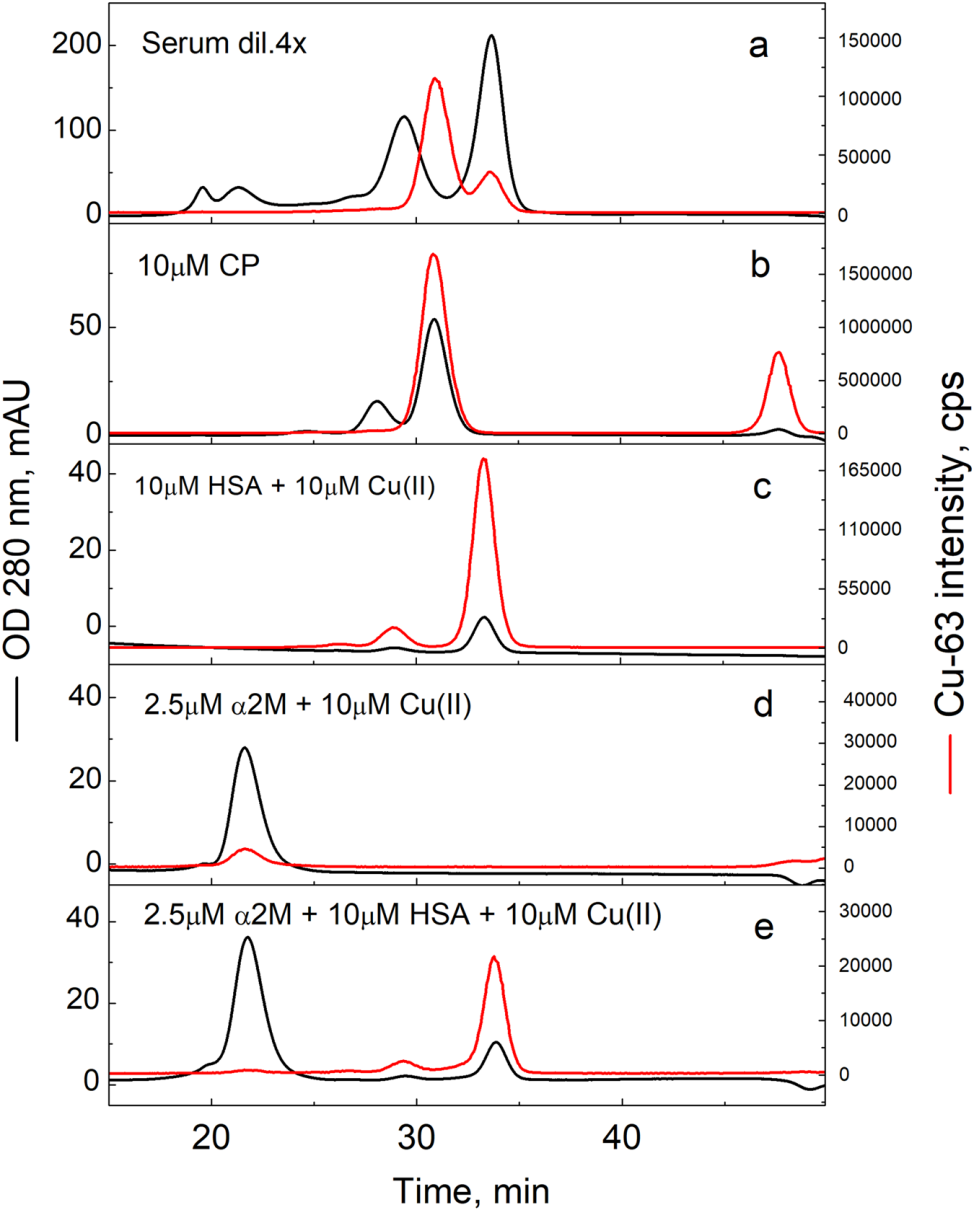
Separation of human serum proteins by SEC. 4 x diluted human serum (a), 10 μM Cu•CP (**b**), 10 μM HSA plus 10 μM Cu(II) (**c**), 2.5 μM α2M tetramer plus 10 μM Cu(II) (**d**), 10 μM HSA, 2.5 μM α2M tetramer plus 10 μM Cu(II) (**e**). Conditions: incubation buffer 50 mM HEPES, 50 mM NaCl, pH 7.4; LC-ICP MS: column - 10 × 300 mm Superdex 200; elution buffer 200 mM NH_4_NO_3_, pH 7.4; flow rate 0.4 ml/min; injection volume 10 μl; UV at 280 nm (left axis and black lines) and Cu-63 (right axis and red line), monitored by ICP MS. Figure was created by program Origin 9 Pro (https://www.originlab.com/).

The experiments were carried out with two preparations of normal (“slow form”) human plasma α2M (from Sigma and Athens Research) as well as with the so-called “fast form” of α2M from Athens Research. The “slow form” of α2M, represents more than 99% of total α2M in blood plasma and possesses the ability to bind and inhibit proteases. The “fast form” of α2M arises through a conformational change caused by entrapment of a protease in the α2M bait region, or chemical cleavage of an internal thiol ester bond located near the bait region^17^. “Fast form” of α2M represents only 0.17–0.7% of the total α2M in blood plasma and is rapidly taken up by the liver. Our LC-ICP MS experiments on Sephadex G25 column showed that both forms of α2M neither contained copper nor were able to bind added Cu(II) ions essentially (Supplementary Information, Figure S2). We tested Cu(II) binding of normal α2M also by ultrafiltration with 10 kD cut-off membranes and confirmed that α2M had only marginal ability (<5%) for retention of Cu(II) ions during ultrafiltration. These results suggest that α2M from human plasma does not bind Cu(II) ions with a biologically significant (e.g. µM or higher) affinity. We also performed a metal competition experiment with α2M and HSA by LC-ICP MS using a Superdex 200 (10 × 300 mm) column, which can separate α2M and HSA peaks. When an equimolar concentration of Cu(II) was added to the equimolar mixture of normal α2M and HSA, almost all copper was eluted in a single peak corresponding to Cu•HSA, demonstrating that α2M cannot compete for Cu(II) ions with HSA even at equimolar concentration (Fig. 5e).

## Discussion

The affinities of metalloproteins for the metal ions, characterized by *K*_*D*_ values, are the key thermodynamic parameters, which direct the metabolism of metal ions and determine their homeostasis. The metal homeostasis can be understood and described quantitatively only if the affinities of the proteins involved are comparable to each other, e.g. they have been determined absolutely correctly or with the same method and using the same reference compounds to ensure the comparability. The full set of comparable metal-binding affinities is available only for the intracellular Cu(I) proteome^32^. The metal-binding affinities of the individual Cu(II) proteins, determined by different methods vary largely as their accurate estimation is a subject to a number of pitfalls and complicating factors^29^. Here we have applied an unified approach for determination of Cu(II)-binding affinities for three major blood copper proteins with the aim to get reliable and comparable *K*_*D*_ values for individual proteins determining the copper distribution in the blood and also in other extracellular media such as cerebrospinal fluid.

### Binding of Cu(II) ions to human serum albumin

The release of Cu(II) ions from Cu•HSA complex in the presence of strong multidentate ligands such as EDTA and NTA is a slow process. The independence of the Cu•HSA demetallation half-life on the multidentate ligands and their concentrations used suggests that the rate-limiting step of the metal release is the dissociation of Cu(II) ions from NTS of HSA. NTS of HSA is highly dynamic^33^ and no large-scale protein conformational changes or other slow processes are assumed to occur during dissocition. Moreover, the maximal half-life of unassisted dissociation of a complex with *pK*_*D*_ 13, is approx. 30 min that is similar to the observed half-life of Cu•HSA decoppering in the presence of EDTA and NTA, which supports our suggestion. The fast release of copper from Cu•HSA in the presence of His points to a different mechanism of demetallation and involvement of His in the rate-limiting step. Based on spectroscopic studies it was suggested that His forms a ternary complex with Cu•HSA^34^, however, a direct [^14^C]His binding assay by using ultrafiltration showed that His interaction with Cu•HSA could be only transient^8^. Nevertheless, even a transient His•Cu•HSA can act as a ligand-exchange complexes enhancing the rate of copper release. This conclusion is also supported by the catalytic effect of physiological concentrations of His (50 - 100 μM) on the demetallation of Cu•HSA by EDTA observed in the current study. The catalytic effect on metal release from Cu•HSA was specific for His as neither 1 mM Glu nor 0.5 mM Gly did not accelerate the reaction. From the X-ray crystal structure of model peptides (DAHK) it is known that Cu(II) ion in NTS (DAH) is equatorially coordinated by N-terminal amine, two deprotonated peptide bond nitrogens and imidazole of His-3, whereas one apical coordination site is occupied with water^35^. It can be suggested that replacement of this water molecule in Cu•HSA with imidazole from free His facilitates the metal release from the ternary complex formed.

The dissociation constant (*K*_*D*_) value for Cu•HSA complex may substantially depend on many factors including the experimental setup of its determination. First, much attention has to be paid to reaching the equilibrium, which is slow in case of many chelators including NTA. Second, our results demonstrate that the estimated *K*_*D*_ value for Cu•HSA complex depends also on the nature and binding mode of the competing ligand used in metal competition experiments. Using of NTA, which forms 1: 1 complex with Cu(II) in our experimental conditions and showed slow demetallation of Cu•HSA, a *K*_*D*_ value equal to 0.900 ± 0.091 pM (50 mM Hepes, 50 mM NaCl, pH 7.4) was determined. However, titration with His, which forms 2: 1 complex in our experimental conditions and leads to fast demetallation of Cu•HSA, yielded *K*_*D*_ equal to 34.7 ± 4.5 fM (50 mM Hepes, 50 mM NaCl at pH 7.4). The lower *K*_*D*_ value determined from competition with His might arise due to the omitting of the formation of a putative ternary complex between HSA, Cu(II) ion and His in the binding scheme, which should result in higher affinity estimates, since the copper in the ternary complex is found in the HMW peak. Thus, the *K*_*D*_ value determined with NTA should be considered more reliable. This value is very similar to the *K*_*D*_ value 1.0 pM, determined from spectroscopic titration of Cu•HSA with NTA in 100 mM NaCl (pH 7.4)^28^. At the same time a substantially higher *K*_*D*_ value 4.0 pM was obtained in 100 mM HEPES, pH 7.4, which was explained with the formation of ternary HEPES• Cu(II)•NTA complex^28^. We note that in addition to the effect of ternary complex formation there is also contribution from differences in ionic strength and extinction coefficients in two media, which were not considered. In our LC-ICP MS experiments *K*_*D*_ values (determined by NTA and His) in 5 mM HEPES, 50 mM NaCl (data not shown) were similar to those in 50 mM HEPES, 50 mM NaCl, which allows to conclude that HEPES at 50 mM level does not compete with HSA, NTA and His for Cu(II) binding or ternary HEPES complexes are formed with both Cu•HSA and Cu•NTA. At the same time we observed that even a slight decrease in pH has a substantial effect on the HSA demetallation levels, which shows that the usage of buffer is necessary. The *K*_*D*_ values for ionic equilibria can be affected also by ionic strength. Indeed, conducting NTA titration in 5 mM HEPES, pH 7.4 we observed that absence of 50 mM NaCl caused fivefold increase of the *K*_*D*_ for Cu•HSA, which is, however, apparent and should be corrected by using of *K*_*D*_ value for Cu(II)•NTA complex at low ionic strength. To avoid these fluctuations and also for practical reasons it is advisable to keep ionic strength value in metal-binding experiments close to 0.1 M, which is a standard condition, where *K*_*D*_ values for reference Cu(II)•Ligand complexes are normally determined^36^. Thus, there is a 4.4-fold difference between *K*_*D*_ values determined from NTA competition by spectroscopic (100 mM HEPES, pH 7.4)^28^ and LC-ICP MS-based method (50 mM HEPES, 50 mM NaCl, pH 7.4). However, we cannot compare these results directly as kinetic results of copper release and incubation times, necessary for reaching the equilibrium were not presented in the earlier paper and it was only declared that the metal release from Cu•HSA in the presence of NTA is faster than in case of EDTA^28^. Our *K*_*D*_ value of 0.90 pM is comparable also with *K*_*D*_ = 6.61 pM, determined in 30 mM Hepes, 250 mM NaCl (pH = 7.0) with equilibrium dialysis^37^, however, difference could be ascribed to different pH value. Therefore *K*_*D*_ value of 0.90 pM for Cu•HSA determined in the present work could serve as a reference *K*_*D*_ value for Cu•HSA in up to 50 mM HEPES buffer, 50 mM NaCl, pH 7.4, useful for further research. From a methodological point of view our study highlights the need to use a kinetic approach and direct methods for quantification of metallated protein complexes as well as the neccessity for strict control of buffer, ionic strength and pH value during titration with metal-chelating ligands.

### Binding of Cu(II) ions to ceruloplasmin

Our results confirm that at pH 7.4 Cu•CP complex can not be demetallated with high millimolar concentrations of strong Cu(II)-chelating ligands like EDTA or DTPA in a timeframe of up to 20 h, which could be explained with three possibilities: first – the thermodynamic Cu(II)-binding affinity of Cu•CP is much higher as compared to chelators used (for EDTA *K*_*D*_ = 1.26 × 10^−16^ M^30^; for DTPA *K*_*D*_ = 5.0 × 10^−17^ M^38^, both at pH 7.4); secondly - dissociation of Cu(II) ions from Cu•CP complex is extremely slow, i.e. complex is kinetically inert at physiological pH values or third - the Cu•CP complex is thermodynamically very stabile and also kinetically inert. Based on available data it is impossible to distinguish between these three possibilities and therefore it is also impossible to determine or estimate *K*_*D*_ for Cu•CP complex from our results. It is known that in Cu•CP the copper ions are located in three mononuclear and one trinuclear binding sites, which are not exposed to the environment^39^. Such encapsulation of metal ions in the protein interior might hinder their dissociation and exclude formation of ternary ligand exchange complexes leading to kinetical inertness of the Cu•CP complex at pH 7.4. Cu•CP could be demetallated by EDTA at non-physiological pH value (pH = 11) in dose-dependent manner, characteristic for fast binding equilibrium. Such behaviour could most probably be explained with partial opening of protein conformation or accelaration of conformational dynamics at alkaline pH values. Thus, our results confirm earlier conclusions that CP-bound copper ions are practically nonexchangeable at physiological pH values and CP does not participate in the regulation of exchangeable copper levels in the blood^5^.

### Binding of Cu(II) ions to alpha-2 macroglobulin

Our results demonstrated that α2M does not bind Cu(II) ions. In earlier experiments α2M has been shown to bind a substoichiometric amount of Cu(II) ions^18^, which is difficult to interpret in terms of thermodynamics. It has been noticed that purified α2M can be contaminated by HSA^40^ that binds Cu(II) ions, which can be mistakenly interpreted as binding of Cu(II) to α2M. We performed also LC-ICP MS experiments using Superdex 200 column, where peaks of CP, HSA and α2M elute at different timepoints (Fig. 5b–d). The α2M sample was supplemented with equimolar concentration of Cu(II) ions, however, only substoichiometric traces of copper were detected in the peak corresponding to α2M (Fig. 5d), which confirms that α2M cannot bind Cu(II) ions. In the competition experiment with 2.5 µM α2M, 10 µM HSA and 10 µM Cu(II) only HSA was metallated (Fig. 5e). Moreover, only two protein-bound copper peaks, corresponding to the major CP and minor HSA, were detected in case of a pooled human serum sample by similar SEC experiment, whereas practically no copper was detected in the HMW region corresponding to α2M (Fig. 5a).

### Copper equilibrium in blood

The average total concentration of copper in normal human blood serum is 16.7 μM^41^. Our results suggest that copper is distributed only between two principal serum proteins – CP and HSA, which expose different affinity and mechanisms of metal binding. In Cu•CP complex, which constitutes approximately 75% from the total copper pool (our data and^4,5^), the copper ions are bound in protein interior, which makes them kinetically inert and practically nonexchangeable at physiological pH values even in the presence of high millimolar concentrations of powerful Cu(II)-chelating ligands like EDTA or DTPA.

Metal-binding mode of HSA differs from that of CP as the NTS of HSA is highly dynamic and exposed to the environment^33^. Cu(II) binding to this site is reversible, however, characterized by slow unassisted dissociation rate and picomolar affinity (*K*_*D*_ = 0.90 ± 0.09 pM), determined with NTA or fast and His-assisted dissociation and apparent subpicomolar affinity (*K*_*D*_ = 34.7 ± 4.5 fM). Cu(II) bound to HSA constitutes approximately 25% from total blood copper (on average 4.2 μM) and this copper pool could also be in equilibrium with Cu(II) ions bound to free amino acids, primarily His. The outcome of this competition depends from the Cu(II)-binding affinities and concentrations of HSA, Cu•HSA and His according to the following simplified reaction scheme:

1 $$\begin{array}{lll} & {K}_{D} & \\ Cu+\,HSA & \rightleftharpoons  & Cu\bullet HSA\end{array}$$

2 $$\,\begin{array}{lll} & {K}_{D1} & \\ Cu+\,His & \rightleftharpoons  & Cu\bullet His\end{array}$$

3 $$\,\begin{array}{lll} & {K}_{D2} & \\ Cu\bullet His\,+\,His & \rightleftharpoons  & Cu\bullet Hi{s}_{2}\end{array}$$

HSA and His are present in the blood serum at average 650 μM and 75 μM concentration respectively^22,42–44^. Taking into account the *K*_*D*_ = 34.7 fM (*K*_*D*_ determined from competition with His in the present work)*, K*_*D*1_ = 3.7 × 10^−9^ M and *K*_*D*2_ = 4.7 × 10^−7^ M values for Cu(II)-His complexes at pH 7.4 (taken from^31^) it can be estimated that approximately 0.7 nM of Cu(II) are bound to His mainly in the form of Cu•His_2_ complex (Equations are presented in Materials and methods). Thus, His alone can bind in average 0.04% from total copper in the blood serum. However, it should be noted that other free amino acids, which are present in the blood serum at total 3 mM concentration^42^, can also form tight ternary complexes with Cu(II)-His^45^. Thus, LMW copper pool in the blood serum might be composed mainly from Cu(II)-His-Xaa complexes and can reach appr. 0.2% level. This estimate agrees relatively well with the range of 1–2.4% for low-molecular weight copper pool in blood serum determined in seven independent studies by ultrafiltration^10^. Moreover, besides binding of minor fraction of Cu(II) ions, His can also act as a catalyst enhancing the rate of the copper transfer between Cu•HSA and other proteins and cellular destinations, which might be the most important physiological role of His in copper metabolism. The proposed catalytic role of His in Cu(II) release from HSA is in agreement with the finding that HSA inhibits copper transport into liver cells whereas His can specifically mobilize Cu(II) from plasma and facilitate the process^46^.

## Conclusion

Cu(II)-binding affinities for three major blood copper proteins: human serum albumin, ceruloplasmin and alpha-2-macroglobulin, were studied through their competition with a set of low-molecular weight Cu(II)-binding reference ligands (DTPA, EDTA, NTA, His) by using an unified LC-ICP MS-based approach. A reliable *K*_*D*_ value for human serum albumin was determined and it was demonstrated that metallated ceruloplasmin is resistant for copper release by chelators used, whereas alpha-2 macroglobulin does not bind Cu(II) ions. Obtained thermodynamic and kinetic data allow determination of the copper distribution in the human blood and also in other extracellular media like for example cerebrospinal fluid. Results allow detection of disturbances in copper metabolism, characteristic for many diseases and provide a rationale for effective metalloregulation.

## Methods

### Instrumentation

For LC-ICP MS analyses an Agilent Technologies (Santa Clara, USA) Infinity HPLC system, which consisted of 1260 series µ-degasser, 1200 series capillary pump, Micro WPS autosampler and 1200 series MWD VL detector was coupled with Agilent 7800 series ICP-MS instrument (Agilent, USA). For instruments control and data acquisition, ICP-MS MassHunter 4.4 software Version C.01.04 from Agilent was used. ICP MS was operated under following conditions: RF power 1550 W, nebulizer gas flow 1.03 l/min, auxiliary gas flow 0.90 l/min, plasma gas flow 15 l/min, nebulizer type: MicroMist, isotope monitored: Cu-63. A 1 ml gel filtration column, self-filled with HiTrap Desalting resin Sephadex G25 Superfine (Amersham/GE Healthcare, Buckinghamshire, UK), was used for the separation of HMW and LMW pools. Injection volume of 10 ul for HSA and 2 ul for other proteins were used. Separation of pooled plasma and individual copper proteins was conducted using Superdex 200 SEC column (10 × 300 mm) (Amersham Biosciences AB, Uppsala, Sweden) by using injection volume of 40 μl. ICP MS compatible flow rate of 0.4 ml/min was used in all separations. In order to get rid of contaminating metal ions in the buffer, the mobile phase was eluted through the Chelex100 Chelating Ion Exchange resin (Sigma, Merck KGaA, Darmstadt, Germany) prior to liquid chromatographic separation. The demetallation of the SEC columns before each experiment was conducted by injecting of 1 mM EDTA into the columns (injection volumes were the same for all experiments, depending on the column type and size). For ultrafiltration experiments, 0.5 ml Amicon Ultra centrifugal filters with 10 kDa cut-off were used (Merck Millipore Ltd, Ireland).

### Materials

Ultrapure milliQ water with a resistivity of 18.2 MΩ/cm, produced by a Merck Millipore Direct-Q & Direct–Q UV water purification system (Merck KGaA, Darmstadt, Germany), was used for all applications.

Mobile phase for gel filtration was 200 mM NH_4_NO_3_ at pH 7.5, prepared from TraceMetal Grade nitric acid (Fisher Scientific UK Limited, Leicestershire, UK) and ammonium hydroxide 25% solution (Honeywell Fluka, Seelze, Germany), which is compatible with ICP MS.

Following lyophilized proteins were used: human serum albumin (HSA) from Sigma/Merck (Darmstadt, Germany), alpha-2 macroglobulin (α2M) from human plasma (“slow form” and “fast form”) and ceruloplasmin (CP) from human plasma were from Athens Research and Technology (Athens, USA), α2M was purchased also from Sigma/Merck (Darmstadt, Germany).

All protein stock solutions were prepared in milliQ water and further diluted in reaction buffer solution (containing 50 mM HEPES and 50 mM NaCl, pH 7.4) for all experiments. For metallation of HSA and α2M Cu(II)acetate from Sigma (Sigma/Merck KGaA, Darmstadt, Germany) was used. An equimolar concentration of Cu(II)acetate was added to HSA in 50 mM Hepes, 50 mM NaCl, pH 7.4 and the sample was incubated 10 min at room temperature, which is sufficient for Cu•HSA complex formation (in separate kinetic experiment we established that the half-life of Cu(II) binding to HSA is approx. 1.5 min). An equimolar concentration of Cu(II)acetate was added α2M in 50 mM Hepes, 50 mM NaCl, pH 7.4 and sample was incubated for up to 1 h. Following reagents were used: ethylenediaminetetraacetic acid (EDTA, 99.995% trace metal basis) and diethylenetriaminepentaacetic acid (DTPA) from Sigma/Merck (Merck KGaA, Darmstadt, Germany), nitrilotriacetic acid (NTA) and L-amino acids (His, Glu and Gly) from Fluka (Merck KGaA, Darmstadt, Germany). Stock solutions of amino acids were prepared in pure milliQ water whereas stock solutions of EDTA, DTPA and NTA were neutralized with 0.1 M NaOH to pH 7-8 and further diluted into reaction buffer. Mobile phase and reagent solutions were prepared daily before experiment.

Blood serum of anonymous donors was obtained from West-Tallinn Central Hospital (Estonia) and the study approval was obtained from Tallinn Medical Research Ethics Committee. For current research, blood plasma pool from 15 individuals was prepared, in order to eliminate individual variability and exclude possibility for identification of a participants. We confirm that all experiments were performed in accordance with relevant guidelines and regulations.

### SDS PAGE

Commercial protein samples have been analyzed by SDS PAGE performed by the Mini-PROTEAN Tetra System (BioRad) with Laemmli gel (10%T/5%C) consisting of separation and comb gel. Samples were prepared in milliQ water at concentration of 3 mg/ml. Samples (20 µl) were mixed with 5 µl of 5x loading buffer (0.3 M Tris-HCl pH = 6.8, 25% β-mercaptoethanol, 50% glycerol, 10% SDS, 1% bromophenol blue), heated to 95 °C for 5 min, and 15 µl of samples were applied to the gel together with 5 µl of “Thermo Scientific PageRuler Unstained Broad Range Protein Ladder”. Electrophoresis was performed with 25 mM Tris, 192 mM Glycine, 0,1% SDS running buffer for about 1 h (130 V). The gel was stained in Coomassie staining solution for overnight followed by destaining in milliQ water and the results, presented in Figure S3 show, that all commercial proteins used have high purity and are suitable for metal-binding studies.

### Calculations for dissociation constants

First, the titration curves (Figs. 3b and 4b) were fitted to a dose-dependent demetallation curve to determine the ligand concentration where the concentrations of copper ions bound to the protein was decreased by 50%. At that point, the equilibrium concentration of the free copper ions [Cu]_free_, which can be calculated according to Eqn.4^31^ equals to the *K*_*D*_ value of the protein.

4 $${[{Cu}]}_{{free}}=\frac{1}{1+\frac{[{L}]}{{{K}}_{1}}+\frac{{[{L}]}^{2}}{{{K}}_{1}\ast {{K}}_{2}}}\,\ast \,({[{Cu}]}_{{total}}-{[{Cu}]}_{{bound}{to}{protein}})\,$$

In the Eqn.4 L is the concentration of the competing ligand, *K*_1_ and *K*_*2*_ correspond to the dissociation constants of Cu•L and Cu•L_2_ complexes, respectively [Cu]_total_ is the concentration of copper and [Cu]_bound to protein_ is the concentration of the copper bound to the protein at IC50.

### Calculations for concentrations of free Cu(II) ions and Cu(II)•His_2_ complex

The concentration of free Cu(II) ions at equilibrium can be calculated as follows:

5 $${[{Cu}]}_{{free}}=\frac{{{K}}_{{D}}\,\ast \,[{Cu}\bullet {HSA}]}{{[{HSA}]}_{{free}}}$$

[Cu]_free_ in blood serum is calculated from the metallation equilibrium of HSA taking into account the total HSA concentration 650 µM and the concentration of non-CP bound copper available for HSA in blood 4.2 µM. [Cu]_free_ is related to the amount of copper bound to LMW ligands like His according to Eqn. 4 and the concentration of LMW copper is given by:

6 $${[{Cu}]}_{{LMW}}={[{Cu}]}_{{free}}\,\ast \,\left(1+\frac{[{L}]}{{{K}}_{1}}+\frac{{[{L}]}^{2}}{{{K}}_{1}\ast {{K}}_{2}}\right){,}$$

where [L] is the concentration of the competing ligand (His), *K*_*1*_ and *K*_*2*_ correspond to the dissociation constants of CuHis and CuHis_2_ complexes (*K*_*1*_ = 3.7 × 10^−9^ M, *K*_2_ = 4.7 × 10^−7^ M, pH = 7.4^31^). Considering the average concentration of His in blood 75 µM^43^, the concentration of Cu•His complexes (mainly Cu•His_2_) is equal to 0.7 nM. Considering that the total concentration of free amino acids in the blood is approximately 3 mM^45^, then concentration of Cu•His•Xaa ternary complexes in the blood is appr. 28 nM.

## Supplementary information

Supplementary information.
