# Supplementary material for: Copper(II)-binding equilibria in human blood
Source: Sci Rep. 2020 Mar 30;10:5686. doi: 10.1038/s41598-020-62560-4 (PMC7105473; doi:10.1038/s41598-020-62560-4)
Supplement: Supplementary file 1 — Supplementary information. [file 41598_2020_62560_MOESM1_ESM.pdf]

Supplementary Information for

Copper(II)-binding equilibria in human blood

Tiina Kirsipuu, Anna Zadorožnaja, Julia Smirnova, Merlin Friedemann, Thomas Plitz, Vello Tõugu, Peep Palumaa

This file includes:

Figures S1, S2 & S3

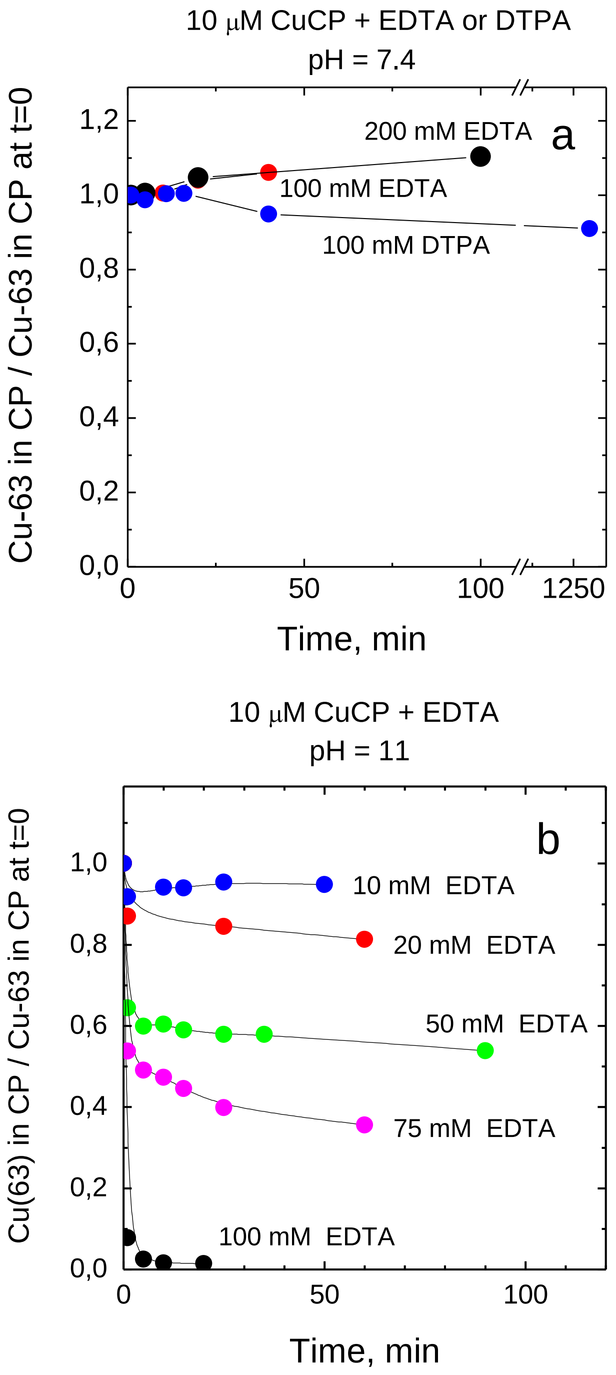

Figure S1. Effect of EDTA and DTPA on metal content in Cu•CP. Relative content of Cu-63 in CP in time in the presence of EDTA and DTPA at pH 7.4 (a) and at pH 11 (b). Conditions: 10 µM Cu•CP, 100 - 200 mM EDTA and 200 mM DTPA; incubation buffer 50 mM Hepes, 50 mM NaCl, pH 7.4; (A); 10 µM Cu•CP, 10 - 100 mM EDTA; incubation buffer 50 mM Hepes, 50 mM NaCl, pH 11; LC-ICP MS: column - 1 ml Sephadex G25 Superfine; elution buffer 200 mM NH_4NO_3, pH 7.4; flow rate 0.4 ml/min; injection volume 10 µl, Cu-63 was monitored by ICP MS. Figure was created by program Origin 9 Pro (https://www.originlab.com/).

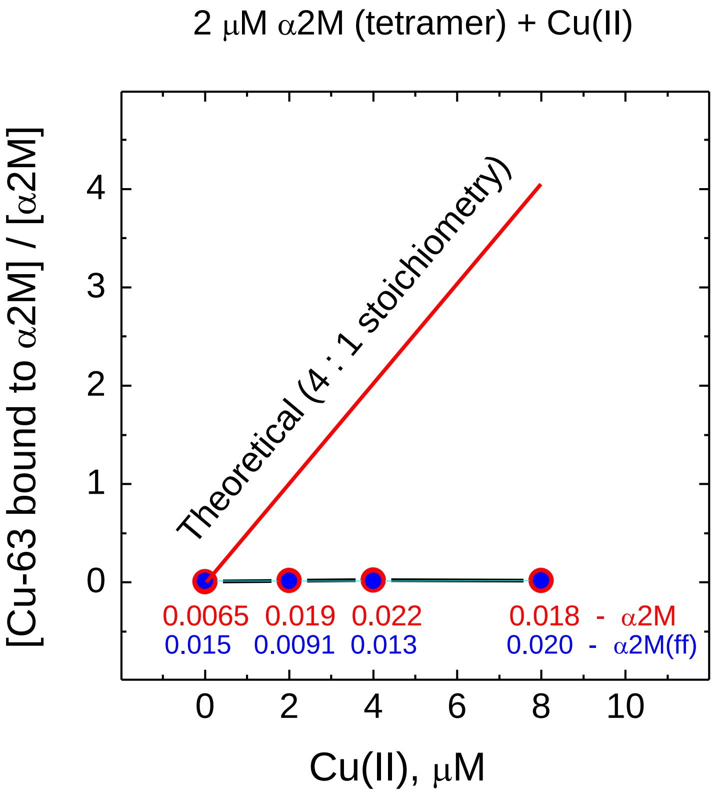

Figure S2. Binding of Cu(II) ions to α2M. Red line – theoretical 4 : 1 binding of Cu(II) to tetrameric α2M), blue line - practical binding of Cu(II) by "normal" (red circles) and "fast form" (blue circles) of α2M. Conditions: 2 μM α2M, 0 – 8 μM Cu(II); incubation buffer 50 mM Hepes, 50 mM NaCl, pH 7.4; LC-ICP MS: column - 1 ml Sephadex G25 Superfine; elution buffer 200 mM NH_4NO_3, pH 7.4; flow rate 0.4 ml/min; injection volume 10 μl, Cu-63 was monitored by ICP MS. Figure was created by program Origin 9 Pro (https://www.originlab.com/).

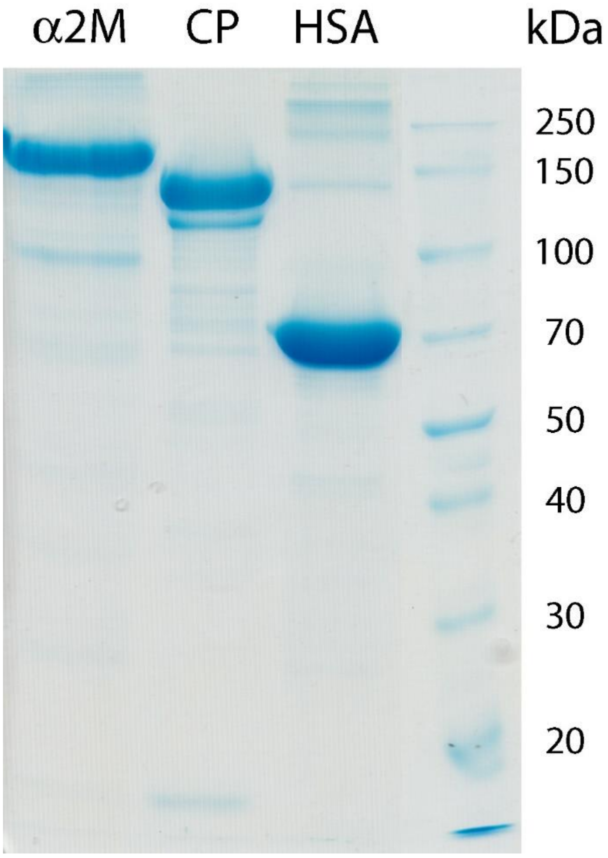

Figure S3. SDS PAGE of commercial HSA, CP and α2M samples with Laemmli gel (10%T/5%C). Protein samples were prepared in milliQ water at concentration of 3 mg/ml. Samples (20 µl) were mixed with 5 µl of 5x loading buffer (0.3M Tris-HCl pH=6.8, 25% β-mercaptoethanol, 50% glycerol, 10% SDS, 1% bromophenol blue), heated to 95ºC for 5 min, and 15 µl of samples were applied to the gel together with 5µl of "Thermo Scientific PageRuler Unstained Broad Range Protein Ladder". Electrophoresis was performed with 25mM Tris, 192 mM Glycine, 0,1% SDS running buffer for about 1 h (130 V). The gel was stained in Coomassie staining solution for overnight followed by destaining in milliQ.
